# Clinical Significance of Ultrasound-Based Hemodynamic Assessment of Extracranial Internal Carotid Artery and Posterior Cerebral Artery in Symptomatic and Angiographic Evolution of Moyamoya Disease: A Preliminary Study

**DOI:** 10.3389/fneur.2021.614749

**Published:** 2021-05-17

**Authors:** Shuai Zheng, Peicong Ge, Zhiyong Shi, Jingzhe Wang, Yi Li, Tengfei Yu, Jinghan Zhang, Hongxia Zhang, Dong Zhang, Wen He

**Affiliations:** ^1^Department of Ultrasound, Beijing Tiantan Hospital, Capital Medical University, Beijing, China; ^2^Department of Neurosurgery, Beijing Tiantan Hospital, Capital Medical University, Beijing, China; ^3^Department of Ultrasound, Tianjin Medical University Cancer Institute and Hospital, Tianjin, China

**Keywords:** moyamoya disease, ultrasound, stroke, extracranial internal carotid artery, posterior cerebral artery

## Abstract

**Objective:** To investigate the hemodynamic changes using ultrasound according to digital subtraction angiography (DSA) findings and explore the association between ultrasound parameters and clinical symptoms of moyamoya disease (MMD).

**Methods:** Hemodynamic parameters of extracranial internal carotid artery (EICA) and posterior cerebral artery (PCA) were evaluated by ultrasound. According to DSA findings, EICA parameters among Suzuki stages (stage I-II, III-IV, and V-VI), and PCA parameters among leptomeningeal system scores (score 0–2, 3–4, and 5–6) were compared, respectively. ROC analysis was performed based on the ultrasound parameters to distinguish stroke from non-stroke patients.

**Results:** Forty patients with MMD were included in our study (16 men; median age, 37 years). The diameter (D), peak systolic velocity (PSV), end diastolic velocity (EDV) and flow volume (FV) of EICA decreased as the Suzuki stage advanced (D: *P* < 0.001; PSV: *P* < 0.001; EDV: *P* < 0.001; FV: *P* < 0.001). The PSV and EDV of PCA increased as the leptomeningeal system scores advanced (PSV: *P* < 0.001; EDV: *P* < 0.001). ROC analysis showed that the area under the curves (AUCs) based on the D and FV of EICA, the PSV and EDV of PCA and their combination were 0.688, 0.670, 0.727, 0.684, and 0.772, respectively, to distinguish stroke from non-stroke patients.

**Conclusions:** Ultrasound parameters were related to Suzuki stages and leptomeningeal system scores. Ultrasound may be useful in predicting the occurrence of stroke in patients with MMD. Future prospective studies with large sample sizes and long-term follow-up are needed to confirm our preliminary findings.

## Introduction

Moyamoya disease (MMD) is a rare disease of unknown etiology, it is characterized by progressive stenosis of the bilateral terminal portions of internal carotid arteries, and their main branches with compensatory abnormal vascular networks at the base of the brain (1). The incidence of moyamoya disease is high in East Asia countries such as Japan and Korea. In Japan, the annual incidence was 0.35 per 100,000. Cerebral ischemia and intracranial hemorrhage due to the rupture of fragile collateral vessels that compensate for ischemia are the main hazards of MMD (2–5). MMD is an important cause of stroke in children and adults. Affected first-degree relatives and those who cannot receive revascularization surgery for a period of time, undergoing imaging at regular intervals can reduce the risk of permanent disability caused by stroke and improve long-term prognosis (6, 7).

Digital subtraction angiography (DSA) plays an important role in guiding the clinical treatment and assessing the prognosis of MMD. Suzuki et al. proposed a conventional criterion for the diagnosis and grading of MMD based on vascular morphology by using DSA (1, 8), however, due to the abundant collateral networks in MMD patients, MMD patients with the same Suzuki stage may have different cerebrovascular reserves and clinical symptoms. Recently, Liu et al. combined the leptomeningeal system from the posterior cerebral artery (PCA) territory to the anterior cerebral artery (ACA) and middle cerebral artery (MCA) territory, which is the most important compensatory system in patients with MMD, and Suzuki stage, proposed a new grading system to better evaluate the clinical characteristics, guide treatment, and predict prognosis (9, 10). However, due to the invasive procedure, high radiation exposure and time consumption of angiography, long-term monitoring has many limitations. Ultrasonography as a non-invasive, economical, and repeatable technique has shown certain value in the diagnosis and prognostic assessment of MMD (11–13). Hong et al. (14) demonstrated that flow volume (FV) of the internal carotid artery (ICA) detected by ultrasound was inversely correlated with Suzuki's grade. Yasuda et al. (15) showed the ratio of the extracranial ICA (EICA) and common carotid artery diameters tended to be lower in symptomatic arteries than in asymptomatic arteries, the ratio decreased as cerebral vasoreactivity decreased. Wang et al. (16) indicated that as the velocity of the PCA decrease, the ischemic lesions spread to a wider range and perfusion levels decreased from good to poor. Based on the above studies, we aim to explore the association between ultrasound parameters, DSA findings and clinical symptoms of patients with MMD. Carotid ultrasound and transcranial color-coded duplex sonography (TCCS) were used to detect hemodynamic changes in the EICA and PCA to realize real-time monitoring of patients with MMD, providing more information for evaluating clinical symptoms in patients with MMD.

## Materials and Methods

### Study Population

**Figure 1:**
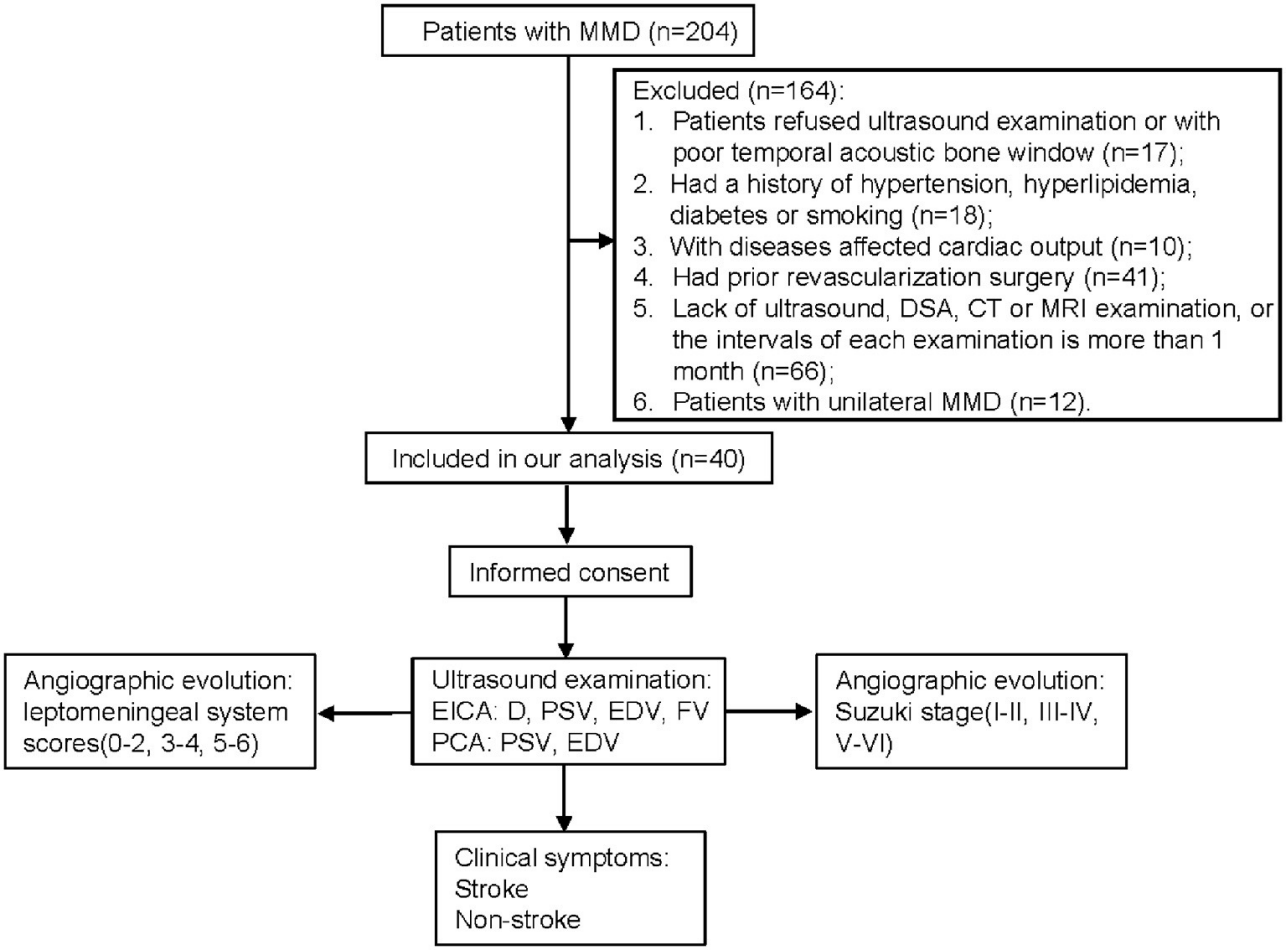
Flow diagram of the study. MMD, moyamoya disease; DSA, digital subtraction angiography; CT, computed tomography; MRI, magnetic resonance imaging; EICA, extracranial internal carotid artery; D, diameter; PSV, peak systolic velocity; EDV, end diastolic velocity; FV, flow volume; PCA, posterior cerebral artery.

We prospectively enrolled consecutive patients with MMD at our institution between July 2019 and February 2020. The inclusion criteria for our study were: (1) patients who were diagnosed with MMD according to the MMD guidelines (17); (2) patients who received ultrasound, DSA, computed tomography (CT) and magnetic resonance imaging (MRI) examinations with intervals of these examinations was <1 month. The exclusion criteria were: (1) patients who were diagnosed with moyamoya syndrome with identified causes (17, 18); (2) patients refused ultrasound examination or with poor temporal acoustic bone window; (3) patients had a history of hypertension, hyperlipidemia, diabetes or smoking; (4) patients with proximal internal carotid artery or vertebrobasilar disease; (5) patients who had diseases that affected cardiac output; (6) MMD patients who had prior revascularization surgery; and (7) patients with unilateral MMD. Finally, a total of 40 patients were included in our study (Figure 1). Informed consent was obtained from all eligible patients (or their parent or legal guardian in the case of children under 16 years), and the study was approved by the Ethics Committee of Beijing Tiantan Hospital, Capital Medical University.

### Angiographic Findings

All 40 patients underwent DSA, including the bilateral ICA, external carotid artery (ECA) and vertebral artery to evaluate collateral flow. Two independent experienced investigators interpreted the images according to the following diagnostic criteria, they were blinded to the clinical data, and any differences in their results were resolved by consensus.

#### Suzuki's Vascular Criteria (1, 7)

Stage I: narrowing of ICA apex; stage II: dilatation of the intracerebral main arteries and initiation of the moyamoya; stage III, narrowing of the MCA and ACA and intensification of the moyamoya; stage IV: occlusion of the ICA extending to the junction of the posterior communicating artery and minimization of the moyamoya, resulting in enlargement of the collateral vessels from the external carotid artery; stage V: disappearance of all the main cerebral arteries and further minimization of the moyamoya; and stage VI: complete disappearance of the siphon of ICA, and disappearance of the moyamoya, resulting in cerebral blood flow supply from the external carotid artery and vertebrobasilar artery systems.

#### Grading Score of the Leptomeningeal System From the PCA Territory to The ACA and MCA Territory

According to the anatomy extent of pial collateral blood (10), the scores of the leptomeningeal system from the PCA territory to the ACA and MCA territory were the sum of the following 3 parts, a score of 0 was assigned if the leptomeningeal anastomoses were absent. (1) Retrograde flow from the parieto-occipital branch of the PCA or posterior pericallosal artery extending to the ACA territory: a score of 1 was assigned if the blood supply extended to the cortical border zone between the ACA and PCA territory; a score of 2 was assigned if the blood supply extended to the central sulcus. (2) A score of 1 was assigned if the anterior temporal branch of the PCA anastomoses to the temporal branch of the MCA. (3) The parieto-occipital branch of PCA anastomoses to the MCA: a score of 1 was assigned if the retrograde flow only extended to superficial vessels (M4 segment of MCA); a score of 2 was assigned if the retrograde flow extended into the Sylvian fissure (M3 segment of MCA); and a score of 3 was assigned if the flow extended to the reconstituted vessels at the distal end of the occlusion (M1 or proximal M2 segments of MCA).

### Clinical Symptoms

According to clinical symptoms, patients were categorized into stroke group and non-stroke group by an experienced research neurologist. The stroke group included patients presented with ischemic stroke and hemorrhagic stroke. Ischemic stroke was defined as a new symptomatic neurologic deterioration lasting at least 24 h that was not caused by a non-ischemic cause, or a new symptomatic neurologic deterioration accompanied by a new cerebral infarction that was not caused by a non-ischemic cause, cerebral infarction was confirmed by MRI. Hemorrhagic stroke was defined as the acute extravasation of blood into the brain parenchyma, cerebral hemorrhage was confirmed by CT (19). The non-stroke group included patients presented with transient ischemic attack (TIA), headache, etc., with no evidence of cerebral infarction and hemorrhage on CT and MRI.

### Ultrasound Examination

All subjects underwent ultrasound examination in the ultrasound department of our hospital by two experienced sonographers. Among them, 20 patients were randomly selected for the intra- and interrater reliability study, the second sonographer performed the same examination immediately after the first one, the first sonographer repeated the examination the next day, and the sonographers were blinded to the clinical data and radiographic findings.

#### Carotid Ultrasound

Carotid ultrasound was performed on a color-coded ultrasound system (EPIQ 7, Philips Medical Systems, Bothell, WA) with a 3–12 MHz linear array probe. The patient remained in a supine position with their head remaining dropped back and tilted to the opposite side slightly. The sonographer adjusted the gain, depth, pulse-repetition frequency and wall filter to the appropriate conditions, the size of the doppler sample volume was adjusted to 1/3–1/4 of the detected vessel, the doppler angle was adjusted to ≤ 60°, and the parameters of the EICA were measured on the two-dimensional longitudinal section at 1–2 cm above the carotid bulb. The following parameters were measured: diameter (D), peak systolic velocity (PSV) and end-diastolic velocity (EDV). Then, the sonographer adjusted the doppler sample volume to the entire width of the vessel, when the signal was stable, the time-averaged mean velocity (TAMV) was measured over a minimum of three cardiac cycles, and the FV was calculated as the product of TAMV and the cross-sectional area (A) of the circular vessel according to the formula FV=TAMV×A=TAMV×[(D/2)^2^×π] (20, 21).

#### Transcranial Color-Coded Duplex Sonography

Transcranial color-coded duplex sonography was performed on a color-coded ultrasound system (EPIQ 7, Philips Medical Systems, Bothell, WA) with a 1.5–3.0 MHz phased array probe. The patient remained in a lateral position, the P2 segment of PCA was examined through a transtemporal window. The sonographer adjusted the gain, pulse-repetition frequency and wall filter to the appropriate conditions, the size of the doppler sample volume was adjusted to 3–5 mm, the depth of insonation for PCA was 60–70 mm, and the doppler angle was adjusted to < 60°, when the signal was stable, the PSV and EDV of PCA were measured.

### Statistical Analysis

Continuous variables were described as median (interquartile range), and categorical variables were described as percentages. The Mann-Whitney U test and Jonckheere-Terpstra test were used for continuous variables. Receiver operating characteristic (ROC) analysis was used to evaluate the discrimination performance of ultrasound parameters in distinguishing stroke from non-stroke patients with MMD. The intra-class correlation coefficient (ICC) was used to assess the intra- and interrater reliability of ultrasound parameters. Statistical analyses were performed using SPSS version 24.0 (IBM Corporation, Armonk, NY). All calculated *P*-values were 2-tailed, and a *P* < 0.05 was considered statistical significance.

## Results

### Patient Characteristics

A total of 40 patients were included in our study, including 16 males and 24 females. All patients had bilateral MMD. The median age of the patients was 37 (28–44) years. According to clinical symptoms, there were 27 patients in the stroke group and 13 patients in the non-stroke group. Suzuki stages were as follows: stage I in 8 hemispheres, stage II in 19 hemispheres, stage III in 35 hemispheres, stage IV in 11 hemispheres, stage V in 6 hemispheres, and stage VI in 1 hemisphere. Grading score of the leptomeningeal system from the PCA territory to the ACA and MCA territory were score 0 in 5 hemispheres, score 1 in 2 hemispheres, score 2 in 12 hemispheres, score 3 in 9 hemispheres, score 4 in 28 hemispheres, score 5 in 21 hemispheres, score 6 in 3 hemispheres (Table 1).

**Table 1:** Baseline characteristics of eligible patients.

**Characteristics**	**Value**
Age (y), median (interquartile range)	37 (28–44)
Sex, male (%)	16 (40.00)
**Clinical symptoms (patients) (%)**	
Stroke	27 (67.50)
Non-stroke	13 (32.50)
**Suzuki stage (hemispheres) (%)**	
I	8 (10.00)
II	19 (23.75)
III	35 (43.75)
IV	11 (13.75)
V	6 (7.50)
VI	1 (1.25)
**Grading score of the leptomeningeal system (hemispheres) (%)**	
0	5 (6.25)
1	2 (2.50)
2	12 (15.00)
3	9 (11.25)
4	28 (35.00)
5	21 (26.25)
6	3 (3.75)

### Association Between Suzuki Stage and Ultrasound Parameters of the EICA in Patients With MMD

**Figure 2:**
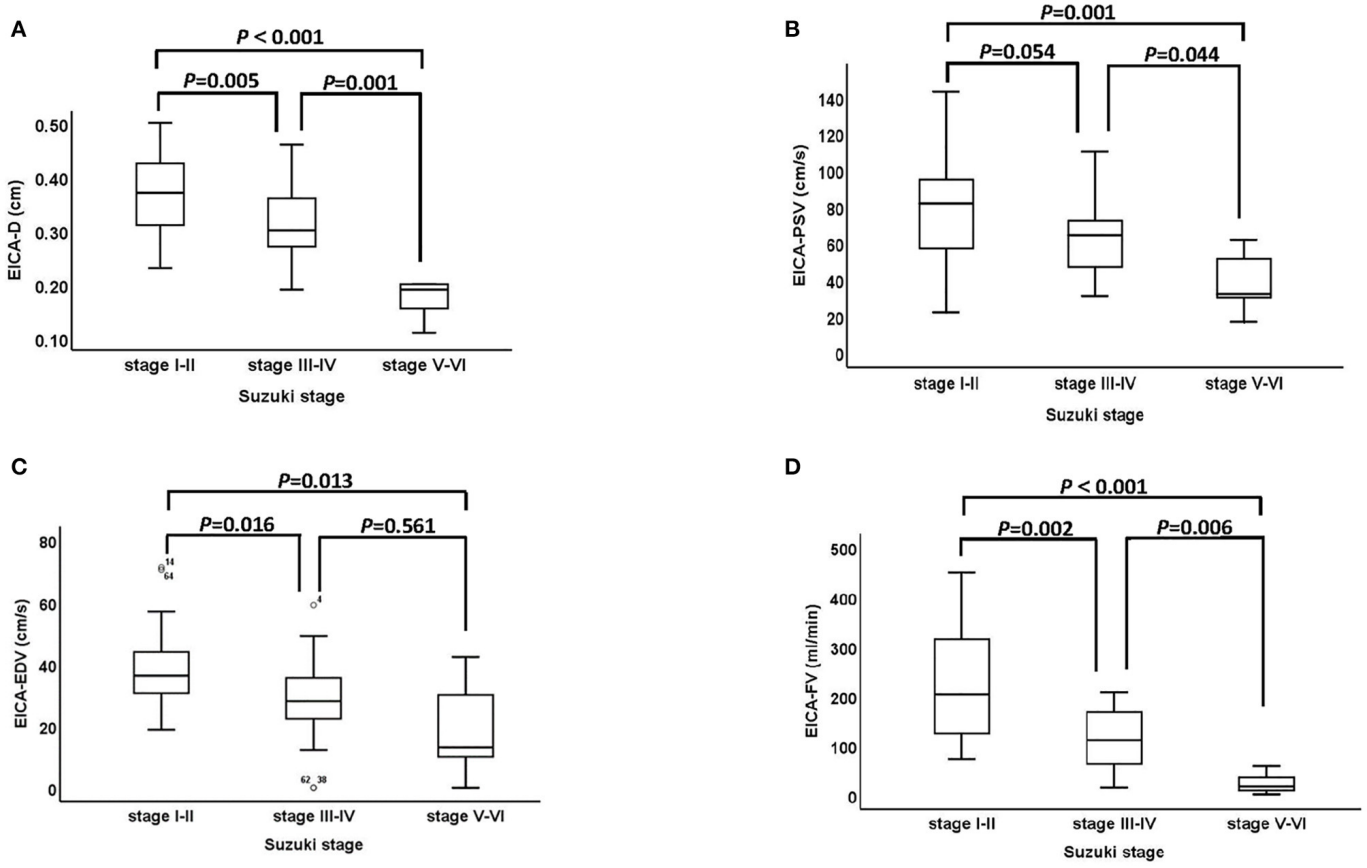
The association between Suzuki stage and ultrasound parameters of the EICA. **(A–D)** The D, PSV, EDV, and FV of EICA between stage I-II, stage III-IV and stage V-VI groups were statistically significant (D: *P* < 0.001, PSV: *P* < 0.001, EDV: *P* < 0.001, FV: *P* < 0.001). EICA, extracranial internal carotid artery; D, diameter; PSV, peak systolic velocity; EDV, end diastolic velocity; FV, flow volume.

**Figure 3:**
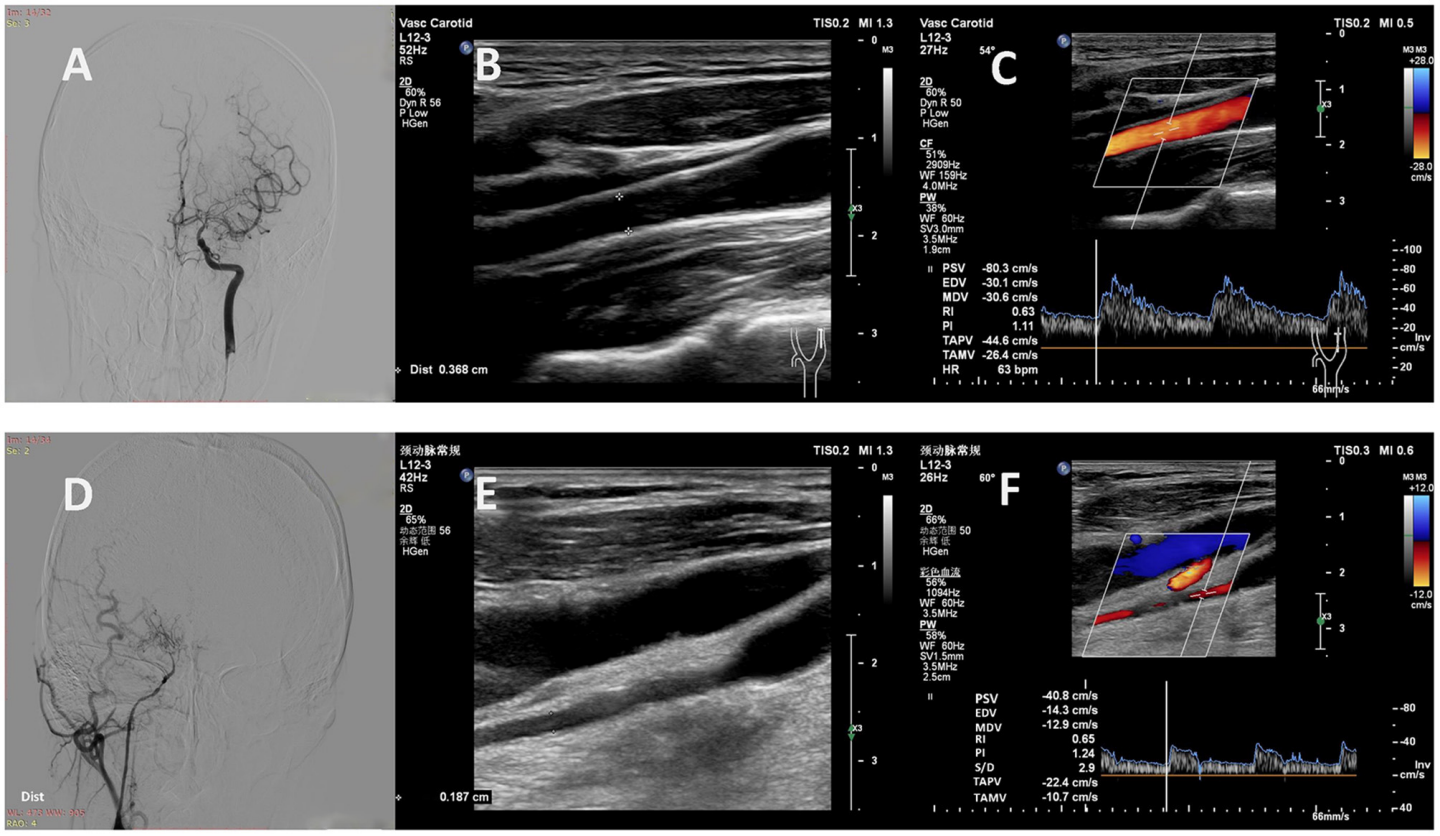
Representative cases. **(A)** A 32-year-old man with MMD had a Suzuki stage II on the left. **(B, C)** Carotid ultrasound showed high values of D (0.37 cm), PSV (80 cm/s), EDV (30 cm/s), TAMV (26 cm/s) and FV (168 ml/min) in the left EICA. **(D)** A 37-year-old woman with MMD had a Suzuki stage V on the right. **(E, F)** Carotid ultrasound showed low values of D (0.19 cm), PSV (41 cm/s), EDV (14 cm/s), TAMV (11 cm/s) and FV (18 ml/min) in the right EICA. MMD, moyamoya disease; D, diameter; PSV, peak systolic velocity; EDV, end diastolic velocity; TAMV, time-averaged mean velocity; FV, flow volume; EICA, extracranial internal carotid artery.

The association between Suzuki stage and ultrasound parameters of the EICA in MMD patients are shown in Figure 2. The D, PSV, EDV and FV of the EICA decreased as the Suzuki stage advanced (D: *P* < 0.001, Figure 2A; PSV: *P* < 0.001, Figure 2B; EDV: *P* < 0.001, Figure 2C; FV: *P* < 0.001, Figure 2D). Representative cases are shown in Figure 3.

### Association Between Grading Score of the Leptomeningeal System From the PCA Territory to the ACA and MCA Territory and Ultrasound Parameters of the PCA in Patients With MMD

**Figure 4:**
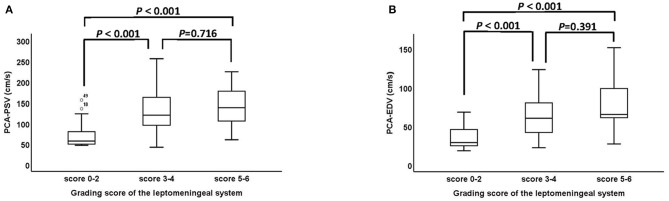
The association between grading score of the leptomeningeal system from the PCA territory to the ACA and MCA territory and ultrasound parameters of the PCA. **(A,B)** The PSV and EDV of PCA between the score 0–2, score 3–4 and score 5–6 groups were statistically significant (PSV: *P* < 0001, EDV: *P* < 0.001). PCA, posterior cerebral artery; ACA, anterior cerebral artery; MCA, middle cerebral artery; PSV, peak systolic velocity; EDV, end diastolic velocity.

**Figure 5:**
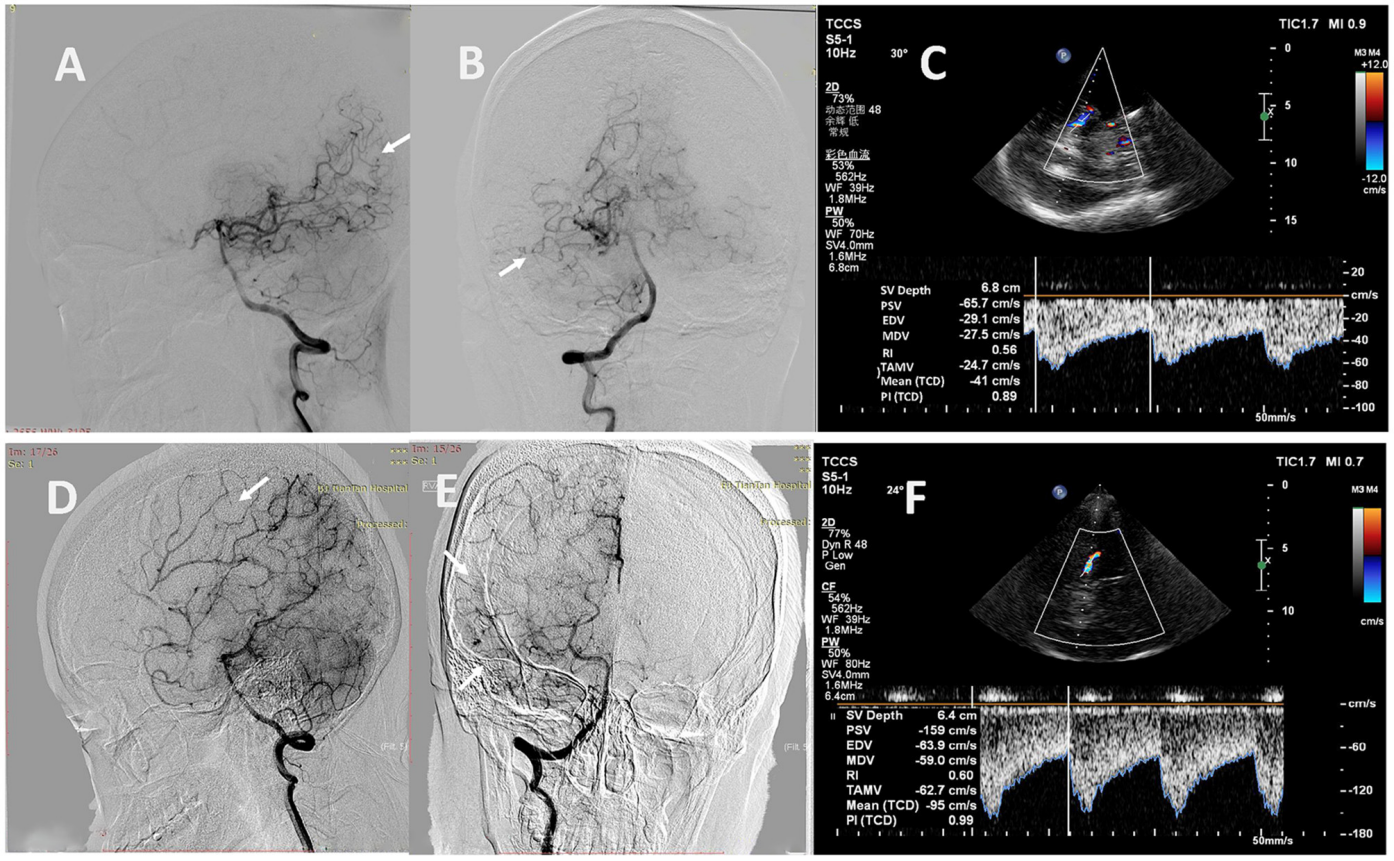
Representative cases. **(A, B)** A 24-year-old man with MMD scored 2 points in the leptomeningeal system on the right (white arrows). **(C)** The TCCS showed low values of PSV (66 cm/s) and EDV (29 cm/s) in the right PCA. **(D, E)** A 33-year-old woman with MMD scored 5 points in the leptomeningeal system on the right (white arrows). **(F)** The TCCS showed high values of PSV (159 cm/s) and EDV (64 cm/s) in the right PCA. MMD, moyamoya disease; TCCS, transcranial color-coded duplex sonography; PSV, peak systolic velocity; EDV, end diastolic velocity; PCA, posterior cerebral artery.

The association between grading score of the leptomeningeal system and ultrasound parameters of the PCA are shown in Figure 4. The PSV and EDV of PCA increased as the scores of the leptomeningeal system from the PCA territory to the ACA and MCA territory advanced (PSV: *P* < 0.001, Figure 4A; EDV: *P* < 0.001, Figure 4B). Representative cases are shown in Figure 5.

### Comparison of Ultrasound Parameters Between Stroke and Non-Stroke Groups of Patients With MMD

The comparison of ultrasound parameters between stroke and non-stroke groups of patients with MMD are shown in Table 2. Compared with the stroke group, the D and FV of EICA were significantly higher in the non-stroke group (D: *P* = 0.007, PSV: *P* = 0.014), the PSV and EDV of PCA were also significantly higher in the non-stroke group (PSV: *P* = 0.001, EDV: *P* = 0.008).

**Table 2:** Comparison of ultrasound parameters between the stroke group and the non-stroke group in patients with MMD.

	**Clinical Symptoms**		
**Characteristics**	**stroke (*n* = 27 patients)**	**Non-stroke (*n* = 13 patients)**	***P-*Value**
**EICA**			
D(cm)	0.30(0.25–0.36)	0.37(0.30–0.41)	0.007
PSV (cm/s)	61.75(40.30–82.13)	65.75(59.43–73.83)	0.334
EDV (cm/s)	30.55(19.83–39.93)	31.70(26.33–36.20)	0.825
FV (ml/min)	103.00(52.82–177.93)	161.36(119.27–228.71)	0.014
**PCA**			
PSV (cm/s)	104.80(62.65–133.50)	150.85(118.75–200.35)	0.001
EDV (cm/s)	51.20(30.03–68.93)	65.50(58.20–97.05)	0.008

*MMD, moyamoya disease; EICA, extracranial internal carotid artery; D, diameter, PSV, peak systolic velocity; EDV, end diastolic velocity; FV, flow volume; PCA, posterior cerebral artery*.

### Diagnostic Value of Ultrasound Parameters to Distinguish Stroke From Non-Stroke Patients With MMD

**Figure 6:**
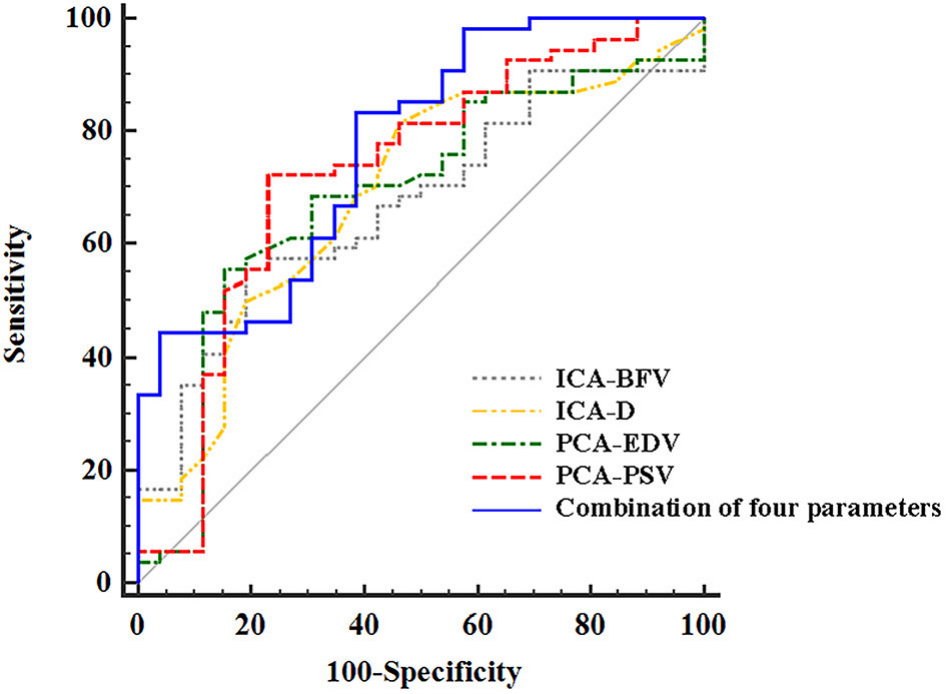
The ROC curves of ultrasound parameters to distinguish stroke from non-stroke patients with MMD. The area under the ROC curves was 0.688 (95% CI, 0.575, 0.787) for the D of EICA, 0.670 (95% CI, 0.556, 0.771) for the FV of EICA, 0.727(95% CI, 0.616, 0.821) for the PSV of PCA, 0.684 (95% CI, 0.571, 0.784) for the EDV of PCA, and 0.772(95% CI, 0.665, 0.858) for the combination of the four parameters. ROC, receiver operating characteristic; MMD, moyamoya disease; EICA-D, diameter of the extracranial internal carotid artery; EICA-FV, flow volume of the extracranial internal carotid artery; PCA-PSV, peak systolic velocity of the posterior cerebral artery; PCA-EDV, end diastolic velocity of the posterior cerebral artery.

ROC analysis was performed based on the D and FV of EICA, the PSV and EDV of PCA and the combination of the four parameters to distinguish stroke from non-stroke patients with MMD (Figure 6). The area under the curves (AUCs) was 0.688 for the D of EICA, 0.670 for the FV of EICA, 0.727 for the PSV of PCA, 0.684 for the EDV of PCA and 0.772 for the combination of the four parameters, with sensitivity and specificity are 81.48% and 53.85%, 55.56% and 80.77%, 72.22% and 76.92%, 55.56% and 84.62%, 83.33% and 61.54%, respectively.

### Intra- and Interrater Reliability of Ultrasound Parameters

We found excellent intrarater reliability for the D of EICA (ICC:0.973), PSV of EICA (ICC:0.939), EDV of EICA (ICC:0.923), FV of EICA (ICC:0.940), PSV of PCA (ICC:0.947) and EDV of PCA (ICC:0.944), and excellent interrater reliability for the D of EICA (ICC:0.971), PSV of EICA (ICC:0.920), EDV of EICA (ICC:0.917), FV of EICA (ICC:0.927), PSV of PCA (ICC:0.945) and EDV of PCA (ICC:0.941).

## Discussion

In this study, we investigated the hemodynamic changes of the EICA and PCA using ultrasound according to the DSA findings of Suzuki stage and grading score of leptomeningeal system from the PCA territory to the ACA and MCA territory in patients with MMD. The D, PSV, EDV, and FV of EICA decreased as the Suzuki stage advanced, the PSV and EDV of PCA increased as the leptomeningeal system scores advanced. MMD patients who presented with non-stroke symptoms were more likely to obtain higher D and FV of the EICA and higher PSV and EDV of the PCA than those who presented with stroke. Our results suggest that ultrasound parameters are related to DSA findings, and detection of ultrasound parameters might be useful in evaluating the clinical symptoms of patients with MMD.

At present, DSA is the gold standard for the diagnosis of MMD, however, DSA is invasive, radiative and can even cause serious complications. For people who cannot undergo DSA examination, MRI combined with magnetic resonance angiography can be used as an alternative method (17), but MRI combined with magnetic resonance angiography is time-consuming and expensive, and neither of these imaging methods can provide hemodynamic information. However, Clinicians expect to dynamically detect the hemodynamic changes in patients with MMD through simple and non-invasive means. The application of these methods in screening and long-term monitoring of MMD are limited. Ultrasonography is a non-invasive, economical, and repeatable technique, that has been used in the diagnosis of MMD, detecting the patency of bypass vessels, and evaluating postoperative hemodynamic changes (22–24).

The histopathological change in the involved artery in MMD is eccentrically laminated thickening of the intracranial artery, as the disease advances, fibrocellular intimal thickening involves the EICA (25–27). Although most researchers have focused on the intracranial portions of the ICA and their branches in MMD, considering the histopathology aspects, MMD causes extracranial stenosis of the proximal portion of the ICA in some cases, the so-called bottleneck sign, which is a typical vascular finding of MMD (27). Yasuda et al. (15) reported that the bottleneck sign began to appear in patients with Suzuki stage III or higher. Our results demonstrated that as the Suzuki stage advanced, the D of EICA decreased. MMD is characterized by progressive stenosis of the bilateral terminal portions of internal carotid arteries, and their main branches, resulting in increased resistance in the distal vessel and decreased velocity and blood flow volume in the proximal vessel. Ruan et al. (28) showed that the time-averaged mean flow velocity of ICA in MMD patients was lower than that in normal controls, and the resistance index was higher than that in normal controls. Hong et al. (14) indicated that Suzuki's grade was inversely correlated with the FV of ICA. Our findings seemed to be consistent with previous studies, we found that as the Suzuki stage advanced, the PSV, EDV and FV of EICA decreased.

As MMD progresses, blood flow decreases in the anterior circulation, and patients may have TIA, headache even stroke. To sustain adequate cerebral perfusion, PCA could develop collateral branches to compensate for the ischemic brain, and the leptomeningeal system from the PCA plays an important role in supplying the ischemic cortex of the MCA and ACA territories. Liu et al. (10) proposed a new grading system for assessing the collateral circulation of MMD patients, according to the anatomic extent of collateral circulation from the PCA territory to the ACA and MCA territory, the grading score of the leptomeningeal system was scored from 0 to 6. As collateral circulation mainly comes from the P2 segment of PCA, in our study, we selected the P2 segment to measure ultrasound parameters. We found that the low-speed blood flow of the PCA was more common in low-score groups. In contrary, in high-score groups, high-speed blood flow of the PCA was more easily detected. The reason was that the high velocity could provide enough blood flow for the collateral circulation. When the P1 segment of PCA was involved, a low-velocity and low-resistance blood flow signal of the P2 segment was noted, and the low-velocity in P2 segment could not supply enough blood flow for the establishment of collateral circulation (16). Therefore, the more abundant collateral circulation formed by the PCA, the higher flow velocity of the P2 segment we detected.

The main symptoms of MMD are stroke and TIA. Hypoperfusion increases the susceptibility to ischemia, hemodynamic abnormalities are the main mechanism of ischemic stroke (10, 29). Hemorrhagic stroke is a deleterious consequence of compensatory mechanisms in response to ischemia (7). As progressive narrowing of the ICA, poor collateral circulation, rupture of fragile, dilated moyamoya vessels under unusually increased hemodynamic stress is the main cause of cerebral hemorrhage (30). For those who do not pay enough attention to TIA and pediatric patients who cannot accurately describe their TIA symptoms, delayed diagnosis and treatment could increase the risk of permanent disability due to stroke. The association between ultrasound parameters and clinical symptoms has rarely been reported. We assumed that the blood flow in the EICA reflects the blood supply of anterior circulation. Our findings have confirmed our hypothesis, a higher Suzuki stage represents a reduction blood flow in the ICA, indicating intracranial shrinkage of the anterior circulation, which is a risk factor for stroke. According to our study, the FV of EICA were significantly correlated with the clinical symptoms, patients who presented with stroke were more likely to have less FV in the EICA, but those who presented with non-stroke symptoms were more likely to have more FV in EICA. PCA is the main pathway of collateral circulation in patients with MMD, and plays an important role in the compensation of cerebral blood flow when principal conduits are insufficient. Successful compensatory collateralization is considered a preventive measure against stroke in patients with MMD (31). Wang et al. (16) indicated that as the velocity of the PCA decrease, the ischemic lesions spread to a wider range from the ICA to PCA territory and perfusion levels decreased from good to poor perfusion. Our study was coincident with the results of previous study. In our study, we explored the association between ultrasound parameters of the PCA and clinical symptoms of patients with MMD. We found that the increased velocity of PCA results in good collateral circulation could better prevent the occurrence of stroke. Our results showed that compared with the non-stroke group, patients in the stroke group presented a lower velocity of the PCA. In our study, we used ultrasound parameters of the EICA and PCA to assess stroke in patients with MMD. As a result, the combination of ICA and PCA parameters was found to be superior to each single parameter for evaluating stroke in patients with MMD. Our results indicated that ultrasound parameters are related to clinical symptoms.

Our study had some limitations. First, because of the relatively small sample size, patients were only divided into stroke and non-stroke groups according to their clinical symptoms. For patients with MMD, hemorrhagic stroke is a deleterious consequence of compensatory mechanisms in response to ischemia, so we didn't further classify the stroke group into ischemic stroke and hemorrhagic stroke groups. Second, in this study, we only investigated the hemodynamics of the EICA and PCA in patients with MMD, although PCA is the main pathway of collateral circulation in MMD and plays an important role in the compensation of cerebral blood flow, transdural collaterals from the ECA can also supply the ischemic brain. As ECA has many branches, the hemodynamic changes in one or two branches have little effect on the trunk, therefore, in this study, we did not study the parameters of the ECA (1, 32). Third, the present study was a single-center study with a small sample size, we did not perform ultrasound examination on patients with MMD before stroke. Therefore, prospective long-term follow-up studies with a large sample size are needed to confirm our findings.

## Conclusions

Our results suggested that the DSA findings of Suzuki stage and scores of the leptomeningeal system from the PCA territory to the ACA and MCA territory are related to EICA and PCA ultrasound parameters, respectively. Ultrasonography can be used for preliminary screening and periodic monitoring of patients with MMD, detection of ultrasound parameters might be useful in predicting the occurrence of stroke in patients with MMD. Future prospective studies with large sample sizes and long-term follow-up are needed to confirm our findings.

## Data Availability Statement

The raw data supporting the conclusions of this article will be made available by the authors, without undue reservation.

## Ethics Statement

The studies involving human participants were reviewed and approved by Institutional Review Board (IRB) of Beijing Tiantan Hospital, Capital Medical University. Written informed consent to participate in this study was provided by the participants' legal guardian/next of kin. Written informed consent was obtained from the individual(s), and minor(s)' legal guardian/next of kin, for the publication of any potentially identifiable images or data included in this article.

## Author Contributions

SZ and PG: Conception and design. SZ, PG, ZS, JW, YL, and JZ: Acquisition of data. SZ and PG: Analysis and interpretation of data. SZ: Drafting the article. All authors: Critically revising the article and Reviewed submitted version of manuscript. WH and DZ: Approved the final version of the manuscript on behalf of all authors. TY, HZ, DZ, and WH: Study supervision.

## Conflict of Interest

The authors declare that the research was conducted in the absence of any commercial or financial relationships that could be construed as a potential conflict of interest.
